# Analysis of gyrobianisotropic media effect on the input impedance, field distribution and mutual coupling of a printed dipole antenna

**DOI:** 10.1038/s41598-022-13410-y

**Published:** 2022-06-20

**Authors:** Mohamed Lamine Bouknia, Chemseddine Zebiri, Djamel Sayad, Issa Elfergani, Mohammad Matin, Mohammad Alibakhshikenari, Abdullah G. Alharbi, Yim Fun Hu, Raed Abd-Alhameed, Jonathan Rodriguez, Francisco Falcone, Ernesto Limiti

**Affiliations:** 1 Laboratoire d’Electronique de Puissance et Commande Industrielle (LEPCI), Department of Electronics, University of Ferhat Abbas, Sétif -1, 19000 Sétif, Algeria; 2 Laboratoire d’Electrotechnique de Skikda (LES), University 20 Aout 1955-Skikda, 21000 Skikda, Algeria; 3 Instituto de Telecomunicações, Campus Universitário de Santiago, 3810-193 Aveiro, Portugal; 4 School of Engineering and Informatics, University of Bradford, Bradford, BD7 1DP UK; 5 Department of Electrical and Computer Engineering, North South University (NSU), Dhaka, 1229 Bangladesh; 6 Department of Signal Theory and Communications, Universidad Carlos III de Madrid, 28911 Leganés, Madrid Spain; 7 Department of Electrical Engineering, Faculty of Engineering, Jouf University, Sakaka, 42421 Saudi Arabia; 8 Electric, Electronic and Communication Engineering Department, Public University of Navarre, 31006 Pamplona, Spain; 9 Institute of Smart Cities, Public University of Navarre, 31006 Pamplona, Spain; 10 Electronic Engineering Department, University of Rome “Tor Vergata”, Via del Politecnico 1, 00133 Rome, Italy

**Keywords:** Electrical and electronic engineering, Electronics, photonics and device physics, Engineering

## Abstract

In this paper, we present an analytical study for the investigation of the effects of the magnetoelectric elements of a reciprocal and nonreciprocal bianisotropic grounded substrate on the input impedance, resonant length of a dipole antenna as well as on the mutual coupling between two element printed dipole array in three configuration geometries: broadside, collinear and echelon printed on the same material. This study examines also the effect of the considered bianisotropic medium on the electric and magnetic field distributions that has been less addressed in the literature for antenna structures. Computations are based on the numerical resolution, using the spectral method of moments, of the integral equation developed through the mathematical derivation of the appropriate spectral Green’s functions of the studied dipole configuration. Original results, for chiral, achiral, Tellegen and general bi-anisotropic media cases, are obtained and discussed with the electric and magnetic field distributions for a better understanding and interpretation. These interesting results can serve as a stepping stone for further works to attract more attention to the reciprocal and non-reciprocal Tellgen media in-depth studies.

## Introduction

With the advancement of technology and the development of new communication systems, it has become increasingly obvious to use microwave and optical planar structures in order to address the challenge of miniaturization of electronic devices. The characteristics and features of microstrip antenna attract researchers to applying them in modern wireless communication applications such as Wi-Fi, GSM, GPS, RFID, ISM systems and wireless sensors^1–6^, since they are simple, small, inexpensive and easy to mount and integrate with microwave monolithic integrated circuits and highly suitable for antenna array technologies. Despite the remarkable progress that have witnessed planar structures technology in recent years, there is still some related features that have not yet been investigated. Further efforts have to be deployed to properly characterize these electronic components in terms of size as well as in terms of used materials to enhance their overall performances.

Recently, as material sciences have significantly advanced, the artificial mediums, such as chiral and general bianisotropic materials, have gained increased interest from researchers and industrials for their unusual and exciting properties^7–21^. In^22^, a theoretical study for investigating the electromagnetic field distributions and input impedance of a dipole antenna printed on a uniaxial anisotropic medium is presented. In^23^, the mutual coupling of two dipole antennas, printed on an anisotropic substrate, is studied for three different configurations: broadband, linear gradient and gradient after the evaluation of the input impedance. It is shown that surface waves increase the mutual coupling in a fixed linear arrangement of the printed dipoles and contribute to the cross-coupling. This also shows that the complex media present a great potential in the design of innovative microwave components^22,23^.

Chiral antenna structures have been dealt with using different techniques in literature. In^15^ and^16^, microstrip antenna structures and multi-element-strip chiral metamaterial antenna arrays are considered, respectively by employing the Cauchy singularity integral equations where the antenna input impedance, for different types of chiral mediums is investigated. In^16^, the authors reported that using a chiral metamaterial substrate presents potential solutions for mutual coupling reduction between radiators in multi-element strip antenna arrays. In^15^, the effect of chiral substrates, based on left-side elements, on a two-element antenna array input impedance is presented, where a decrease in the quality factor and a shortening effect are noticed. In^18^, a study presents a mathematical model and proposes a method for the electrodynamic investigation of a chiral metamaterial substrate-based microstrip antenna with mirror-asymmetric left-handed conductive spiral inclusions. A characterization of the input impedance is described, where the authors showed that using a chiral substrate causes a shift of the resonant frequency and an important decrease in the quality factor which indicates the possibility of improving the mass-dimensional characteristics of a chiral microstrip antenna. In^19^, a mathematical model of a microstrip antenna implanted on a bi-isotropic chiral substrate is presented based on of the singular integral equation. It is stated that such an antenna radiates elliptically polarized waves, shows an asymmetric radiation pattern and exhibits a lower Q-factor compared to isotropic antennas. Moreover, using a chiral substrate improves the electrodynamics features and reduces the antenna overall size. In^20^, a metamaterial microstrip antenna-based MIMO system is studied. It is stated that the use of such substrates reduces the mutual coupling between the emitters and the use of bi-isotropic chiral substrates in microstrip antennas with fractal strips improves their characteristics.

The works in^15–20,24–26^ have treated only the isotropic and bi-isotropic chiral medium cases, where the authors the input impedance and mutual coupling of single and multilayer dipole antennas are investigated.

In the present paper, we present an analytical study of a dipole antenna printed on a substrate with a gyro-bi-anisotropy where the magneto-electric elements of the constitutive parameters are complex valued. Particularly, the effect of the complex elements on the input impedance, resonant length and mutual coupling of two printed dipoles and hence, antenna performance according to the collinear, echelon and broadside configurations. The study extends our previous our works^10,22,23^, where only anisotropic and bi-isotropic medium cases were treated. It is based on a spectral theoretical formulation and a numerical solution technique using the moments method in the spectral domain; a method that is well known in the analysis of microwave planar structures^7,27^.

This paper consists in two parts. The first part, “Analytical formulation and Method of solution” sections, concerns the analytical evaluation of the complex wave equations in a general bianisotropic medium, Green’s function and the electromagnetic fields expressions related to the printed dipole structure. New mathematical formulations are developed and a brief detail on the method of resolution is presented. The second part, “Numerical results” Section, gathers the results of five cases of gyro-bianisotropic media according to different values of the magneto-electric element. This section, depending on the cases to be treated, is subdivided into five main subsections: The effect of the magneto-electric element on the input impedance, its effect on the coupling between two-element dipole array, the effect of the substrate thickness on the coupling, the effect of the magneto-electric element on the fields distributions and the advantageous combined effect of reciprocal chiral and reciprocal Tellegen elements on the input impedance and mutual coupling with a brief overview table summarizing the main results. In this part, the components of the electromagnetic fields components have been plotted using a calculation code developed in Matlab to show the effect of the complex medium on these components.

## Analytical formulation

The magnetoelectric tensors of the bianisotropic layer material express the coupling between the magnetic and electric fields. The corresponding constitutive relations of a general complex bianisotropic medium are expressed, in their general form, by^7,8,28,29^:

1a $$\vec{D} = \left[ {\upvarepsilon } \right]\vec{E} + \sqrt {\varepsilon_{0} \mu_{0} } \left[ {\upeta } \right]\vec{H}$$

1b $$\vec{B} = \left[ {\upmu } \right]\vec{H} + \sqrt {\varepsilon_{0} \mu_{0} } \left[ {\upxi } \right]\vec{E}$$

where ε is the permittivity, μ is the permeability, ξ and η are the magnetoelectric parameters expressing the coupling between the magnetic and electric fields. In general, for bianisotropic mediums, all the parameters are 3 × 3 tensors.

**Figure 1:**
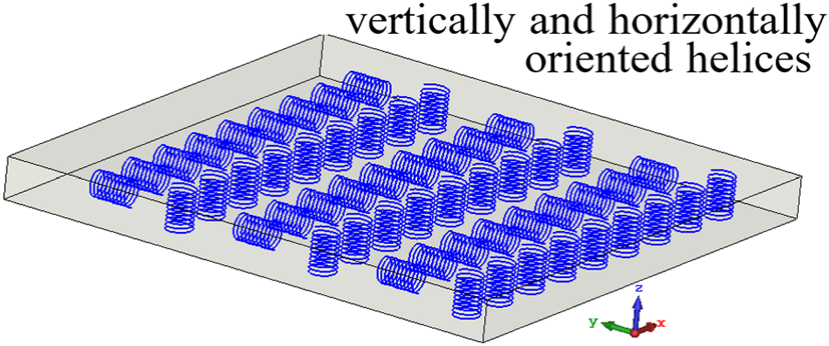
Bi-isotropic substrate^20^.

**Figure 2:**
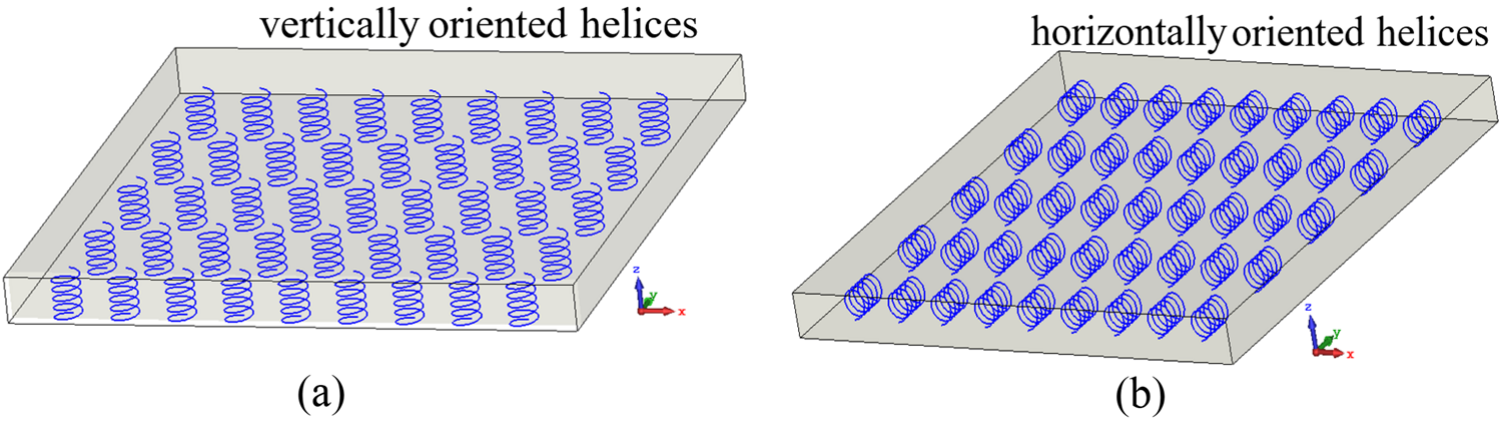
Bi-anisotropic chiral substrates (**a**): vertically and (**b**): horizontally oriented helices^20^.

Chiral materials can be realized using a bi-isotropic medium based on randomly distributed right-and left-handed helices (Fig. 1) or bianisotropic based on orderly distributed right-and left-handed helices. Several configurations of these structures were presented in^20^, in particular, the structures with vertically or horizontally oriented helices (spirals) as illustrated in Fig. 2a,b, respectively.

**Figure 3:**
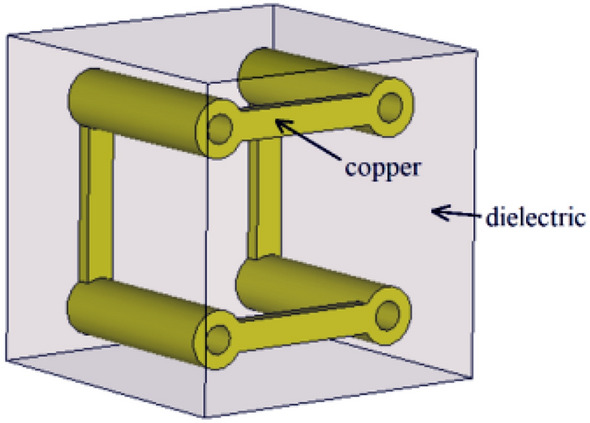
Unit cell of the bi-anisotropic metamaterial medium described by Eq. 2. The copper is inserted in a dielectric medium with a relative permittivity ε_r_ = 2.2.^28^.

In^28^, another type of a bianisotropic metamaterial is designed using Split Ring Resonators (SRRs), schematically shown by its unit cell depicted in Fig. 3. It consists of a pair of orthogonal SRRs. The split ring acts as a LC circuit, where the loop and gap are equivalent to an inductor and a capacitor, respectively. The bianisotropic property can be described by the fields and current distribution for a z-polarized incident wave, as reported in the literature^28^.

In^21^, the authors reported that the Tellegen material response exhibits non-reciprocal possibilities in gainless or lossless media. Suitable numerical methods were developed for computing photonic eigen-modes for designing and characterizing topological order based on Tellegen metacrystals. Strong Tellegen responses can be stimulated in metamaterials composed of magnetic ferrites associated with bianisotropic elements, and it would be simple to implement this proposal experimentally in the microwave domain^21^. Artificial media, such as chiral and SRRs, can also be used to design such systems.

In^30^, the authors investigated the scattering of electromagnetic waves from bounded moving media. They explained the phenomenon of the intraluminal regime of double transmission of waves downstream through a stationary interface between regular medium and moving media. The refractive index and impedance relations for bulk moving media were derived in the subluminal and intraluminal regimes.

In the present work, we consider for study a complex bianisotropic medium characterized by 3 × 3 permittivity, permeability and magnetoelectric tensors. Assuming that the coupling between the magnetic and electric fields only exists in the x–y plane. These tensors have the following forms:

2a $$\left[ {\upvarepsilon } \right] = \varepsilon_{0} \left[ {\begin{array}{*{20}c} {\varepsilon_{t} } & 0 & 0 \\ 0 & {\varepsilon_{t} } & 0 \\ 0 & 0 & {\varepsilon_{z} } \\ \end{array} } \right]$$

2b $$\left[ {\upmu } \right] = \mu_{0} \left[ {\begin{array}{*{20}c} {\mu_{t} } & 0 & 0 \\ 0 & {\mu_{t} } & 0 \\ 0 & 0 & {\mu_{z} } \\ \end{array} } \right]$$

2c $$\left[ {\upxi } \right] = \left[ { - \begin{array}{*{20}c} 0 & {\left( {\chi_{xy}^{{}} + j\xi_{xy}^{{}} } \right)} & 0 \\ {\left( {\chi_{xy}^{{}} + j\xi_{xy}^{{}} } \right)} & 0 & 0 \\ 0 & 0 & 0 \\ \end{array} } \right]$$

2d $$\left[ {\upeta } \right] = \left[ {\begin{array}{*{20}c} 0 & {\left( {\varsigma_{xy}^{{}} + j\eta_{xy}^{{}} } \right)} & 0 \\ { - \left( {\varsigma_{xy}^{{}} + j\eta_{xy}^{{}} } \right)} & 0 & 0 \\ 0 & 0 & 0 \\ \end{array} } \right]$$

Generally, the magnetoelectric coupling effects can be classified as reciprocal or non-reciprocal. In reciprocal media, as can be easily derived from the Lorentz reciprocity theorem, the permittivity and permeability dyadics are symmetric, and the two magnetoelectric dyadic coefficients are related^31^ as $\left[ {\upeta } \right]$ = − $\left[ {\upxi } \right]^{T}$, only one magnetoelectric dyadic is sufficient to describe the nonreciprocal coupling. Indeed, one of the two dyadics is the transpose of the other $\left[ {\upeta } \right]$ = $\left[ {\upxi } \right]^{T}$^31^.

The non-reciprocity parameters $\chi_{xy}^{{}}$ and $\varsigma_{xy}^{{}}$ are needed to model natural magnetoelectric effect which occurs, for example, in some ferromagnetic and anti-ferromagnetic crystals. Recently, it has been suggested how such media can be realized as artificial composites for microwave applications^32,33^. Because these tensors are real valued, the Tellegen response breaks time reversal symmetry, distinguishing it from other bianisotropic electromagnetic responses (such as SRRs and chiral meta-molecules), which do not^21,34,35^.

The $j\xi_{xy}^{{}}$ and $j\eta_{xy}^{{}}$ in some conditions are responsible for chiral effects in isotropic Pasteur media. As is commonly accepted, we use the name chirality parameter for the coupling $j\xi_{xy}^{{}}$ and $j\eta_{xy}^{{}}$ for anisotropic reciprocal media, too^32,33^.

In what follows, we investigate the following five cases according to the conditions of consideration^28–33^, which are:

- $$\left[ {\upxi } \right] = - \left[ {\upeta } \right]^{T} = \left[ {\upeta } \right] = \left[ { - \begin{array}{*{20}c} 0 & {j\xi_{xy}^{{}} } & 0 \\ {j\xi_{xy}^{{}} } & 0 & 0 \\ 0 & 0 & 0 \\ \end{array} } \right]$$ (reciprocal chiral)
- $$\left[ {\upxi } \right] = \left[ {\upeta } \right]^{T} = - \left[ {\upeta } \right] = \left[ { - \begin{array}{*{20}c} 0 & {j\xi_{xy}^{{}} } & 0 \\ {j\xi_{xy}^{{}} } & 0 & 0 \\ 0 & 0 & 0 \\ \end{array} } \right]$$ (non-reciprocal achiral)
- $$\left[ {\upxi } \right] = - \left[ {\upeta } \right]^{T} = \left[ {\upeta } \right] = \left[ { - \begin{array}{*{20}c} 0 & {\chi_{xy}^{{}} } & 0 \\ {\chi_{xy}^{{}} } & 0 & 0 \\ 0 & 0 & 0 \\ \end{array} } \right]$$ (reciprocal Tellegen 1st case)
- $$\left[ {\upxi } \right] = \left[ {\upeta } \right]^{T} = - \left[ {\upeta } \right] = \left[ { - \begin{array}{*{20}c} 0 & {\chi_{xy}^{{}} } & 0 \\ {\chi_{xy}^{{}} } & 0 & 0 \\ 0 & 0 & 0 \\ \end{array} } \right]$$ (non-reciprocal Tellegen 2nd case)
- $$\left[ {\upxi } \right] = - \left[ {\upeta } \right]^{T} = \left[ {\upeta } \right] = \left[ { - \begin{array}{*{20}c} 0 & {\left( {\chi_{xy}^{{}} + j\xi_{xy}^{{}} } \right)} & 0 \\ {\left( {\chi_{xy}^{{}} + j\xi_{xy}^{{}} } \right)} & 0 & 0 \\ 0 & 0 & 0 \\ \end{array} } \right]$$ (reciprocal complex bianisotropic)

**Figure 4:**
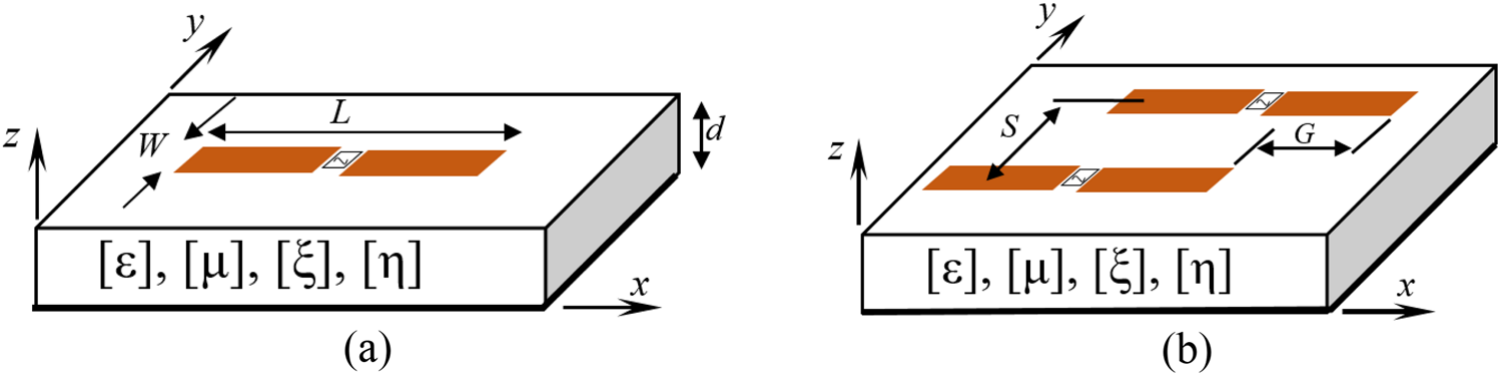
Geometries of (**a**): Printed dipole and (**b**): Two-element dipole array.

The general planar dipole antenna geometry and the associated coordinate system, with the optical axis *oz* as direction of propagation, are illustrated in Fig. 4. The herein studied planar structure is based on a complex bianisotropic grounded substrate. The presented configuration is used to investigate the effect of bianisotropy on the input impedance of the printed dipole (Fig. 4a) and to evaluate the mutual coupling between two-dipole antenna array (Fig. 4b).

In this case study, a realistic bianisotropic material design is considered that supports both TE and TM surface modes. This material can be realized with complex imaginary-valued elements of the constitutive parameters. This is done by inserting a periodic set of inclusions in a dielectric substrate^9^. A realistic experimental model of this bianisotropic metamaterial is presented in^9,28^. The simplest cases of this medium have been studied as a substrate of a dipole antenna by Sayad et al.^26^, where the magnetoelectric elements were equal and purely imaginary.

3 $$\left[ {\upxi } \right] = \left[ {\upeta } \right] = \left[ { - \begin{array}{*{20}c} 0 & { + j\xi_{xy}^{{}} } & 0 \\ {j\xi_{xy}^{{}} } & 0 & 0 \\ 0 & 0 & 0 \\ \end{array} } \right]$$

The results show that the chirality parameter serves as an additional parameter that could be utilized to control or adjust the input impedance for bandwidth improvement and miniaturization of the antenna size. The case of non-equal magnetoelectric elements was studied by Zebiri et al. in^12^.

4a $$\left[ {\upxi } \right] = j\left[ { - \begin{array}{*{20}c} 0 & { + \xi_{xy}^{{}} } & 0 \\ {\xi_{xy}^{{}} } & 0 & 0 \\ 0 & 0 & 0 \\ \end{array} } \right]$$

4b $$\left[ {\upeta } \right] = j\left[ { - \begin{array}{*{20}c} 0 & { + {\upeta }_{xy}^{{}} } & 0 \\ {{\upeta }_{xy}^{{}} } & 0 & 0 \\ 0 & 0 & 0 \\ \end{array} } \right]$$

In^12^, the results showed that the contribution of the magnetoelectric elements *ξ* and *η* in the calculation of the input impedance takes the average form of $\tfrac{1}{2}\left( {\xi + \eta } \right)$ in the dyadic Green’s function described by Eq. 20 in ^12^. On the other hand, the gyro-chiral parameter contribution in the electromagnetic field components is denoted by the factor $e_{{}}^{{ - \kappa_{0} \tfrac{1}{2}\left( {\xi - \eta } \right) \cdot z}}$, in Eqs. 9 and 10^12^, that expresses gain or loss, depending on the choice of the two magnetoelectric parameters *ξ* and *η*.

In^8,11–14,26^, only cases of media with imaginary valued magnetoelectric elements have been investigated, *i.e.,* the case of a reciprocal chiral media $\left( {\left[ {\upxi } \right] = - \left[ {\upeta } \right]^{T} } \right)$. In this work, we will examine the more complex issue, taking into consideration reciprocity and non-reciprocity for complex valued magnetoeclectic element (chiral, achiral, Tellegen and general bi-anisotropic cases). The present work will carry out this novelty of cases that have never been investigated before. The first result which should be taken into consideration is that these media have a significant potential which must be thoroughly studied.

The expected waves propagating in a grounded dielectric slab are surface wave modes which are either TE or TM with respect to the interface normal^36^. We assume that the propagation is along the + *z* direction with *e*^*−jβz*^ as a factor of propagation. The longitudinal electromagnetic field components *E*_*z*_ and *H*_*z*_ are found to satisfy two decoupled homogeneous second-degree differential wave equations:

5a $$\frac{{\partial^{2} \tilde{E}_{z} }}{{\partial z^{2} }} - \Gamma_{1} \frac{{\partial \tilde{E}_{z} }}{\partial z} + \Gamma_{2} \tilde{E}_{z} = 0$$

5b $$\frac{{\partial^{2} \tilde{H}_{z} }}{{\partial z^{2} }} - \Gamma_{1} \frac{{\partial \tilde{H}_{z} }}{\partial z} + \Gamma_{3} \tilde{H}_{z} = 0$$

The particularity of this medium is described by the extra term $\Gamma_{1} \frac{{\partial \tilde{E}_{z} }}{\partial z}$ in the second-order differential wave equation. This additional term can be interpreted by a loss or a gain in the amplitude of the electromagnetic fields^12^, which is reminiscent of the Schrodinger equation for an electron in presence of a magnetic potential^21^.

For $\left[ {\upxi } \right] = \left[ {\upeta } \right]$, $\Gamma_{1} = 0$, we meet the case of reciprocal bianisotropic media. It is confirmed that the non-reciprocity contributes to the appearance of the $\Gamma_{1} \ne 0$ and which can be interpreted by the presence of a magnetic moment^21^.

5c $$\Gamma_{1} = j\kappa_{0} \left( {\varsigma_{xy}^{{}} - \chi_{xy}^{{}} + j\left( {\eta_{xy}^{{}} - \xi_{xy}^{{}} } \right)} \right)$$

5d $$\Gamma_{2} = \left( {\left( {\kappa_{0}^{2} \varepsilon_{t} \mu_{t} - \frac{{\varepsilon_{t} }}{{\varepsilon_{z} }}\left( {\alpha^{2} + \beta^{2} } \right)} \right) + \kappa_{0}^{2} \left( {\chi_{xy}^{{}} + j\xi_{xy}^{{}} } \right)\left( {\varsigma_{xy}^{{}} + j\eta_{xy}^{{}} } \right)} \right)$$

5e $$\Gamma_{3} = \left( {\left( {\kappa_{0}^{2} \varepsilon_{t} \mu_{t} - \frac{{\mu_{t} }}{{\mu_{z} }}\left( {\alpha^{2} + \beta^{2} } \right)} \right) + \kappa_{0}^{2} \left( {\chi_{xy}^{{}} + j\xi_{xy}^{{}} } \right)\left( {\varsigma_{xy}^{{}} + j\eta_{xy}^{{}} } \right)} \right)$$

## Method of solution

Because there are two distinct regions: dielectric (region 1) and air (region 2), we must separately define the fields components in these two regions and then match the tangential fields on the interface air-dielectric. Solving the two differential Eqs. (2a) and (2b) for $\tilde{E}_{z}$ and $\tilde{H}_{z}$ in region 1, gives:

6a $$\tilde{E}_{z} \left( {\gamma_{e} ,z} \right) = e^{{\kappa_{0} \kappa_{c} z}} e^{{j\kappa_{t} \kappa_{0} z}} \left( {A_{e} \cosh \left( {\gamma_{e} z} \right) + B_{e} \sinh \left( {\gamma_{e} z} \right)} \right)$$

6b $$\tilde{H}_{z} \left( {\gamma_{h} ,z} \right) = e^{{\kappa_{0} \kappa_{c} z}} e^{{j\kappa_{t} \kappa_{0} z}} \left( {A_{h} \sinh \left( {\gamma_{h} z} \right) + B_{h} \cosh \left( {\gamma_{h} z} \right)} \right)$$

where $A_{e}$, $B_{e}$, $A_{h}$ and $B_{h}$ are complex constants and

6c $$\kappa_{c} = \frac{{\left( {\xi_{xy} - \eta_{xy} } \right)}}{2}$$

6d $$\kappa_{t} = \frac{{\left( {\varsigma_{xy} - \chi_{xy} } \right)}}{2}$$

6e $$\gamma_{e}^{2} = \left( {\frac{{\varepsilon_{t} }}{{\varepsilon_{z} }}\left( {\alpha^{2} + \beta^{2} } \right) - \kappa_{0}^{2} \varepsilon_{t} \mu_{t} } \right) - \left( {\frac{{\kappa_{0}^{{}} }}{2}\left( {\varsigma_{xy}^{{}} + \chi_{xy}^{{}} + j\left( {\eta_{xy}^{{}} + \xi_{xy}^{{}} } \right)} \right)} \right)^{2}$$

6f $$\gamma_{h}^{2} = \left( {\frac{{\mu_{t} }}{{\mu_{z} }}\left( {\alpha^{2} + \beta^{2} } \right) - \kappa_{0}^{2} \varepsilon_{t} \mu_{t} } \right) - \left( {\frac{{\kappa_{0}^{{}} }}{2}\left( {\varsigma_{xy}^{{}} + \chi_{xy}^{{}} + j\left( {\eta_{xy}^{{}} + \xi_{xy}^{{}} } \right)} \right)} \right)^{2}$$

In the absence of the Tellegen parameter ($$\varsigma_{xy}^{{}} = \chi_{xy}^{{}} = 0$$) the expressions above are the same as those found in^12^.Thus we find that the solution for a plane EM wave in a bianisotropic (lossy or lossless) medium consists of a nonreciprocal z^+^-directed propagating wave and a z^–^-directed one with $$e^{{\kappa_{0} \kappa_{c} z}} e^{{j\kappa_{t} \kappa_{0} z}}$$. For the chiral, this term will be $$e^{{\kappa_{0} \kappa_{c} z}}$$.There may be a gain in one direction and a loss in the other. The non-reciprocal medium (Tellegen) contributes by a phase $$e^{{j\kappa_{t} \kappa_{0} z}}$$ in the solution which must be deeply examined in future works. However, these two traveling waves no longer have a constant amplitude as they move, but rather they decay exponentially with the traveled distance, as indicated by the $$e^{ - \alpha z}$$ term in the z^+^-directed wave and the $$e^{\alpha z}$$ term for the z-directed one. The solution resembles that of a lossy dielectric medium only in one direction and just for one medium ($$\eta_{xy}^{{}} = - \xi_{xy}^{{}}$$$$\varsigma_{xy}^{{}} = - \chi_{xy}^{{}}$$). This is a very interesting feature that has to be well considered. Under these conditions, the medium behaves like an isotropic dielectric with the presence of the term $$e^{{\kappa_{0} \kappa_{c} z}} e^{{j\kappa_{t} \kappa_{0} z}}$$.

In the air region, the field components decay with respect to z, for which, we assume the following expressions:

7a $$\tilde{E}_{z} \left( {\gamma_{0} ,z} \right) = C_{e} e^{{ - \gamma_{0} \left( {z - d} \right)}}$$

7b $$\tilde{H}_{z} \left( {\gamma_{0} ,z} \right) = C_{h} e^{{ - \gamma_{0} \left( {z - d} \right)}}$$

where

7c $$\gamma_{0}^{{}} = \sqrt {\left( {\alpha^{2} + \beta^{2} } \right) - \kappa_{0}^{2} }$$

$C_{e}$ and $C_{h}$ are complex constants.

The application of the appropriate boundary conditions at z = 0 and z = d allows the determination of the complex constants $A_{e}$, $B_{e}$, $C_{e}$, $A_{h}$, $B_{h}$ and $C_{h}$ that appear in the electromagnetic field component expressions in both regions^26^.

Algebraic development of the derived mathematical expressions leads to the formulation of the estimated electric field at the interface air-dielectric between the two regions with respect to the current densities $\tilde{J}_{x}$ and $\tilde{J}_{y}$. The spectral Green's tensor is derived satisfying the following system of equations^12,26^.

8a $$\tilde{E}_{x} = \tilde{G}_{xx} \tilde{J}_{x} + \tilde{G}_{xy} \tilde{J}_{y}$$

8b $$\tilde{E}_{y} = \tilde{G}_{yx} \tilde{J}_{x} + \tilde{G}_{yy} \tilde{J}_{y}$$

where $\tilde{J}_{x}$ and $\tilde{J}_{y}$ are the Fourier transforms of the current densities on the conducting strips.

In the analysis of narrow dipoles configurations, the herein considered structure, the cross-current density in the *y*-direction is commonly ignored, as it is assumed that the width of the dipole is negligible^26^. Consequently, $\tilde{G}_{xx}$ is the only presented Green’s function, since the others are not involved in the calculations. For this bianisotropic medium, $\tilde{G}_{xx}$ is derived and it is given by:

9a $$G_{xx} = \frac{ - j}{{\omega \varepsilon_{0} \left( {\alpha^{2} + \beta^{2} } \right)}}\left[ {\frac{{\alpha^{2} \gamma_{0} \left( {\gamma_{e}^{2} + \gamma_{c}^{2} } \right)}}{{\gamma_{0} \varepsilon_{t} \gamma_{e} \coth \left( {\gamma_{e} d} \right) + \left( {\left( {\gamma_{e}^{2} + \gamma_{c}^{2} } \right) - j\gamma_{0} \varepsilon_{t} \gamma_{c}^{{}} } \right)}}} \right.\, - \left. {\frac{{\beta^{2} \kappa_{0}^{2} \mu_{t} }}{{\left( {\gamma_{h} \coth \left( {\gamma_{h} d} \right) + \mu_{t} \gamma_{0} - j\gamma_{c}^{{}} } \right)}}} \right]$$

with

9b $$\gamma_{c}^{2} = \left( {\frac{{\kappa_{0}^{{}} }}{2}\left( {\left( {\varsigma_{xy}^{{}} + \chi_{xy}^{{}} } \right) + j\left( {\eta_{xy}^{{}} + \xi_{xy}^{{}} } \right)} \right)} \right)^{2}$$

The magnetoelectric-depending sub-cases of this general bianisotropic medium can be verified. For $\chi_{xy} = \varsigma_{xy} = 0$, the Green's tensor expression is the same as that found in^12^. For $\left[ {\upxi } \right] = \left[ {\upeta } \right] \ne 0$, we find the expression derived in^26^ and for a dielectric with a uniaxial anisotropy $\left[ {\upxi } \right] = \left[ {\upeta } \right] = 0$, we obtain the same medium and expressions as those treated in^24,25^.

The mathematical manipulation of the resulting equations gives the formulation of the electric field evaluated at the interface air-dielectric in terms of the current densities $\tilde{J}_{x}$ and $\tilde{J}_{y}$. By applying the boundary conditions, the expressions of the x, y and z components of the electric and magnetic fields in the dielectric and air regions can be formulated as follows:

1st region (dielectric):

10a $$\tilde{E}_{x1} \left( {\alpha ,\beta ,z} \right) = j\frac{{e^{{\tfrac{1}{2}\kappa_{0} \kappa_{c} \left( {z - d} \right)}} }}{{\alpha^{2} + \beta^{2} }}\frac{1}{{\omega \varepsilon_{0} }} \times \left( { - \alpha \left( {\gamma_{e}^{2} + \gamma_{c}^{2} } \right)\gamma_{0} {\text{Se}} \times A_{e} + \beta \kappa_{0}^{2} \mu_{t} {\text{Sh}} \times A_{h} } \right)$$

10b $$\tilde{E}_{y1} \left( {\alpha ,\beta ,z} \right) = j\frac{{e^{{\tfrac{1}{2}\kappa_{0} \kappa_{c} \left( {z - d} \right)}} }}{{\alpha^{2} + \beta^{2} }}\frac{1}{{\omega \varepsilon_{0} }}\, \times \left( { - \beta \left( {\gamma_{e}^{2} + \gamma_{c}^{2} } \right)\gamma_{0} {\text{Se}} \times A_{e} - \alpha \kappa_{0}^{2} \mu_{t} {\text{Sh}} \times A_{h} } \right)$$

10c $$\tilde{E}_{z1} \left( {\alpha ,\beta ,z} \right) = - e^{{\tfrac{1}{2}\kappa_{0} \kappa_{c} \left( {z - d} \right)}} \frac{{\gamma_{0} \gamma_{ec}^{{}} \varepsilon_{t} }}{{\omega \varepsilon_{0} \varepsilon_{z} }}{\text{Se}} \times A_{e}$$

10d $$\tilde{H}_{x1} \left( {\alpha ,\beta ,z} \right) = \frac{{e^{{\tfrac{1}{2}\kappa_{0} \kappa_{c} \left( {z - d} \right)}} }}{{\alpha^{2} + \beta^{2} }}\left( {\beta \gamma_{0} \varepsilon_{t} \gamma_{ec}^{{}} {\text{Se}} \times A_{e} - \alpha \gamma_{hc}^{{}} {\text{Sh}} \times A_{h} } \right)$$

10e $$\tilde{H}_{y1} \left( {\alpha ,\beta ,z} \right) = \frac{{e^{{\tfrac{1}{2}\kappa_{0} \kappa_{c} \left( {z - d} \right)}} }}{{\alpha^{2} + \beta^{2} }}\, \times \left( { - \alpha \gamma_{0} \varepsilon_{t} \gamma_{ec}^{{}} {\text{Se}} \times A_{e} - \beta \gamma_{hc}^{{}} {\text{Sh}} \times A_{h} } \right)$$

10f $$\tilde{H}_{z1} \left( {\alpha ,\beta ,z} \right) = je^{{\tfrac{1}{2}\kappa_{0} \kappa_{c} \left( {z - d} \right)}} \frac{{\mu_{t} }}{{\mu_{z} }}{\text{Sh}} \times A_{h}$$

2nd region (air):

11a $$\tilde{E}_{x2} \left( {\alpha ,\beta ,z} \right) = j\frac{{e^{{ - \gamma_{0} \left( {z - d} \right)}} }}{{\alpha^{2} + \beta^{2} }}\frac{1}{{\omega \varepsilon_{0} }}\, \times \left( { - \alpha \gamma_{0} \left( {\gamma_{e}^{2} + \gamma_{c}^{2} } \right)A_{e} + \mu_{t} \beta \kappa_{0}^{2} A_{h} } \right)$$

11b $$\tilde{E}_{y2} \left( {\alpha ,\beta ,z} \right) = j\frac{{e^{{ - \gamma_{0} \left( {z - d} \right)}} }}{{\alpha^{2} + \beta^{2} }}\frac{1}{{\omega \varepsilon_{0} }}\, \times \left( { - \beta \gamma_{0} \left( {\gamma_{e}^{2} + \gamma_{c}^{2} } \right)A_{e} - \mu_{t} \alpha \kappa_{0}^{2} A_{h} } \right)$$

11c $$\tilde{E}_{z2} \left( {\alpha ,\beta ,z} \right) = \frac{{\left( {\gamma_{e}^{2} + \gamma_{c}^{2} } \right)}}{{\omega \varepsilon_{0} }}A_{e} e^{{ - \gamma_{0} \left( {z - d} \right)}}$$

11d $$\tilde{H}_{x2} \left( {\alpha ,\beta ,z} \right) = \frac{{e^{{ - \gamma_{0} \left( {z - d} \right)}} }}{{\alpha^{2} + \beta^{2} }}\left( { - \beta \left( {\gamma_{e}^{2} + \gamma_{c}^{2} } \right)A_{e} + \mu_{t} \alpha \gamma_{0} A_{h} } \right)$$

11e $$\tilde{H}_{y2} \left( {\alpha ,\beta ,z} \right) = \frac{{e^{{ - \gamma_{0} \left( {z - d} \right)}} }}{{\alpha^{2} + \beta^{2} }}\left( {\alpha \left( {\gamma_{e}^{2} + \gamma_{c}^{2} } \right)A_{e} + \beta \mu_{t} \gamma_{0} A_{h} } \right)$$

11f $$\tilde{H}_{z2} \left( {\alpha ,\beta ,z} \right) = j\mu_{t} A_{h} e^{{ - \gamma_{0} \left( {z - d} \right)}}$$

where $\tilde{J}_{x}$ and $\tilde{J}_{y}$ are the Fourier transforms of the current densities, and

12a $$A_{e} = \frac{{\alpha \tilde{J}_{x} + \beta \tilde{J}_{y} }}{{\left( {\gamma_{e}^{2} + \gamma_{c}^{2} + \gamma_{0} \varepsilon_{t} \left( {\gamma_{e} \coth \left( {\gamma_{e} d} \right) - j\gamma_{c}^{{}} } \right)} \right)}}$$

12b $$A_{h} = \frac{{\beta \tilde{J}_{x} - \alpha \tilde{J}_{y} }}{{\left( {\gamma_{h} \coth \left( {\gamma_{e} d} \right) - j\gamma_{c}^{{}} + \gamma_{0} \mu_{t} } \right)}}$$

12c $$\gamma_{ec}^{{}} = \left( {\gamma_{e} \coth \left( {\gamma_{e} d} \right) - j\gamma_{c}^{{}} } \right)$$

12d $$\gamma_{hc}^{{}} = \left( {\gamma_{h} \coth \left( {\gamma_{h} d} \right) - j\gamma_{c}^{{}} } \right)$$

12e $${\text{Se}} = \frac{{\sinh \left( {\gamma_{e} z} \right)}}{{\sinh \left( {\gamma_{e} d} \right)}}$$

12f $${\text{Sh}} = \frac{{\sinh \left( {\gamma_{h} z} \right)}}{{\sinh \left( {\gamma_{h} d} \right)}}$$

In the present analysis, we also aimed to examine the effect of the gyro-bianisotropic medium on the electric and magnetic field distributions that has been less investigated in previous works in the literature. To evaluate the field distributions in the considered bianisotropic medium, the components of the electric and magnetic fields in both regions in the spatial domain are numerically derived using the inverse Fourier transform.

## Numerical results

In this work, we are interested in the investigation of the effect of the gyro-bianisotropic substrate on the input impedance, the resonant length of the dipole and the mutual coupling between two-element printed dipole array along three configurations. The five cases: chiral, achiral, Tellegen and complex bianisotropic mediums, are investigated and the related original results are discussed and commented.

### Validation

Before discussing the results of this study, a validation of the method and the solution technique is undertaken by comparing with studies published in literature.

In order to test the efficiency of the employed method and the accuracy of the solution technique, an initial comparative study is carried out. We have initially considered the isotropic case (*ε*_*t*_ = *ε*_*z*_ = 3.25 and *μ*_*r*_ = 1).

**Figure 5:**
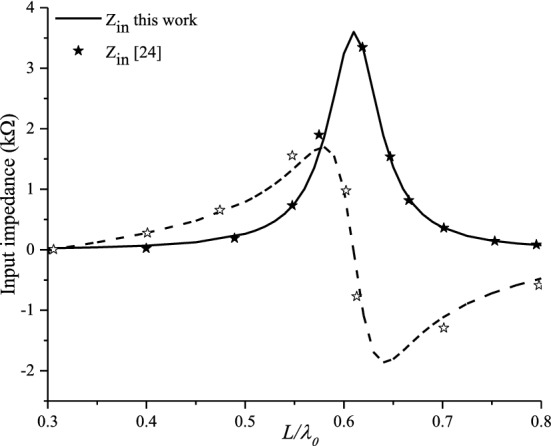
Isotropic input impedance, real and imaginary parts.

**Figure 6:**
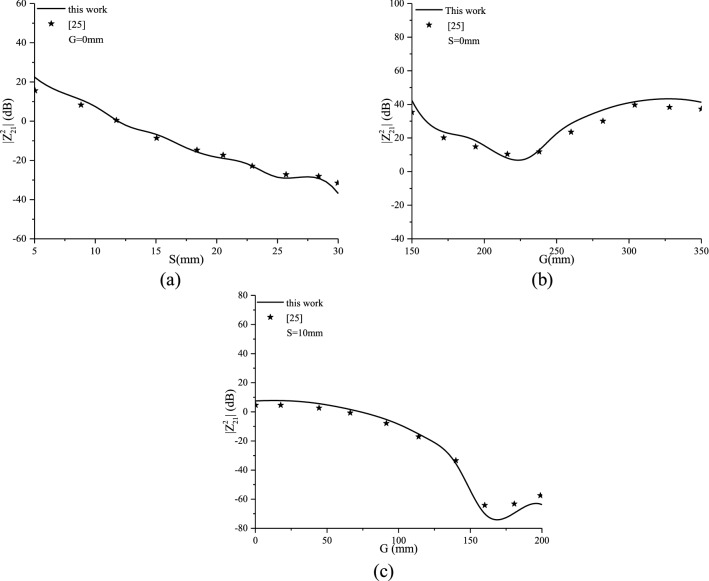
Isotropic mutual coupling of printed dipoles, (**a**): broadside, (**b**): collinear, and (**c**): echelon configurations.

Figure 5 shows the plot of the complex input impedance (real and imaginary parts) of a printed planar dipole of width *W* = 0.0004*λ*_0_ with respect to the normalized length *L/λ*_0_. The dipole is printed on an anisotropic grounded dielectric slab with a thickness of *d* = 0.1060*λ*_0_. Figure 6a–c present the calculated mutual coupling between the printed dipoles for collinear, echelon and broadside configurations, respectively, for L = 150 mm, W = 0.5 mm, f = 500 MHz and d = 1.58 mm.

A comparison representation of the input impedance and mutual coupling configurations for the same configuration parameters used above is given in Figs. 5 and 6. The representation shows a good agreement compared to available data reported in literature^22–25^.

### Effect of the magneto-electric parameters on the input impedance

#### Non-reciprocal achiral (${\upxi }_{{{\text{xy}}}} = - {\upeta }_{{{\text{xy}}}}$)

**Figure 7:**
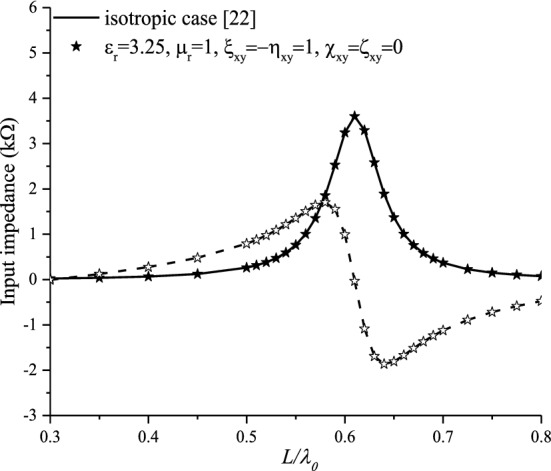
Effect of the non-reciprocal achiral elements (${\upxi }_{{{\text{xy}}}} = - {\upeta }_{{{\text{xy}}}}$) on the input impedance.

Figure 7 shows the input impedance for a non-reciprocal achiral medium case (${\upxi }_{{{\text{xy}}}} = - {\upeta }_{{{\text{xy}}}} = 1$), compared with the isotropic case. No effect is noticed on the input impedance. This can be justified by the electromagnetic fields and Green’s function expressions. In this case (${\upxi }_{{{\text{xy}}}} = - {\upeta }_{{{\text{xy}}}}$), it is noticed that no contribution of the achirality is observed in the expressions of Eqs. 6 and 10.

#### Reciprocal chiral (${\upxi }_{{{\text{xy}}}} = {\upeta }_{{{\text{xy}}}}$)

**Figure 8:**
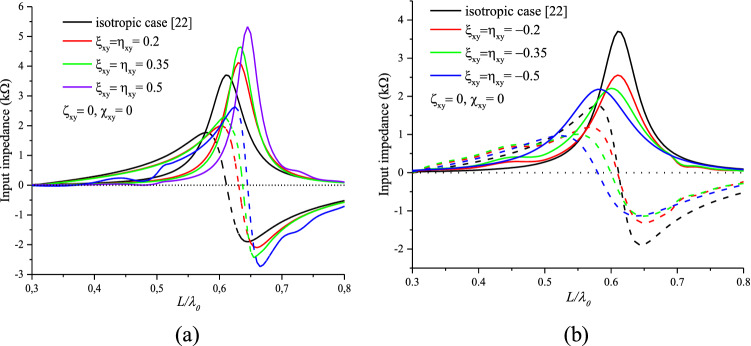
Effects of (**a**): Positive-reciprocal chirality and (**b**): Negative-reciprocal chirality on the input impedance of the printed dipole.

**Figure 9:**
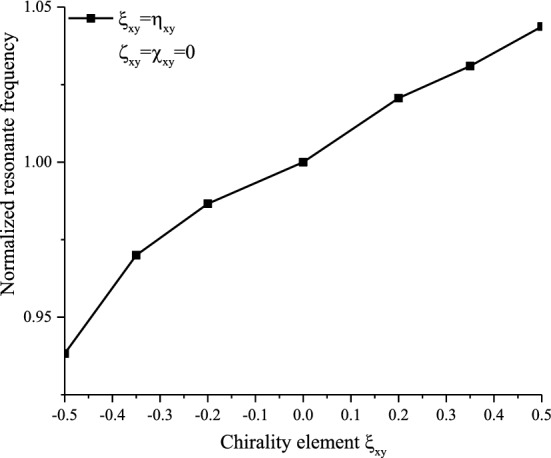
Normalized resonant frequency with reciprocal chiral mediums.

Figure 8a,b illustrate the variation of the input impedance of a reciprocal chiral dipole antenna for different positive and negative values of $\xi_{xy}^{{}}$ and $\eta_{xy}^{{}}$ with a permittivity of $\varepsilon_{r} = 3.25$ and a permeability of $\mu_{r} = 1$, compared to the isotropic case medium. In this case, the effect of the parameters $\xi_{xy}^{{}}$ and $\eta_{xy}^{{}}$ is reciprocal, either on the shape of the input impedance (Fig. 8a,b) or on the resonance frequency (Fig. 9).

The maximum (peak) of the input impedance and the resonance frequency increases with the increasing positive values of $\xi_{xy}^{{}}$ and inversely for negative values. For $\xi_{xy}^{{}}$ = $\eta_{xy}^{{}}$ = 0.5 ($\xi_{xy}^{{}}$ = $\eta_{xy}^{{}}$ = -0.5), an increase (a decrease) of 50% of the input impedance peak is observed.

#### Non-reciprocal Tellegen medium ($\chi_{xy}^{{}} = - \varsigma_{xy}^{{}}$)

**Figure 10:**
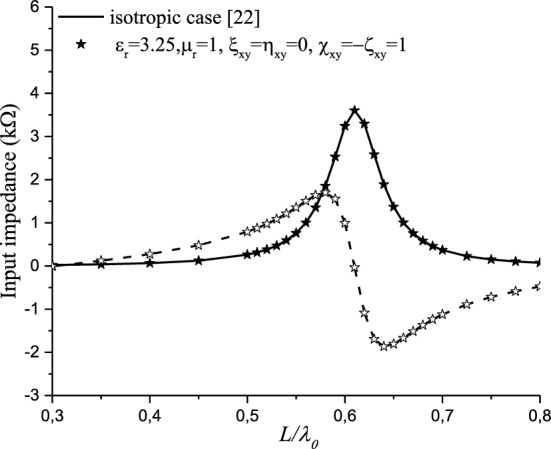
Effect of the non-reciprocal Tellegen medium ($\chi_{xy}^{{}} = - \varsigma_{xy}^{{}}$) on the input impedance.

In this case ($\chi_{xy}^{{}} = - \varsigma_{xy}^{{}}$), similarly to the case of non-reciprocal achiral (${\upxi }_{{{\text{xy}}}} = - {\upeta }_{{{\text{xy}}}} = 1$), it is noticed that no effect of the non-reciprocal Tellegen medium is observed on the input impedance (Fig. 10).

#### Reciprocal Tellegen medium ($\chi_{xy}^{{}}$ = $\varsigma_{xy}^{{}}$)

**Figure 11:**
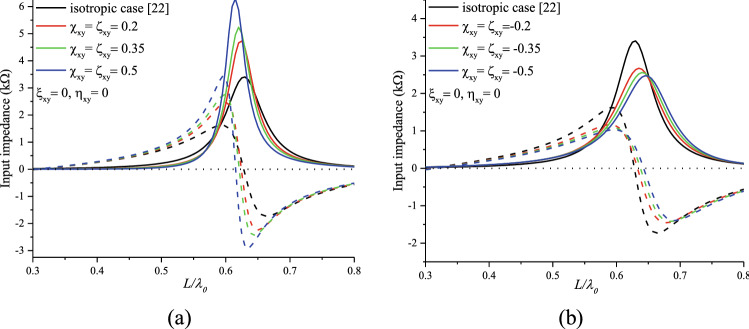
Effects of (**a**): Positive-reciprocal Tellegen and (**b**): negative-reciprocal Tellegen bianisotropic medium on the input impedance of the printed dipole.

Figure 11a,b show an increase in the input impedance amplitude for positive values of $\chi_{xy}^{{}}$ and a decrease for negative ones. However, the resonance points shifted to the left compared with the isotropic case with for positive values (Fig. l1a). As for the negative values, the resonance points moved right (Fig. 11b). We can notice that the Tellegen case affects significantly the input impedance.

**Figure 12:**
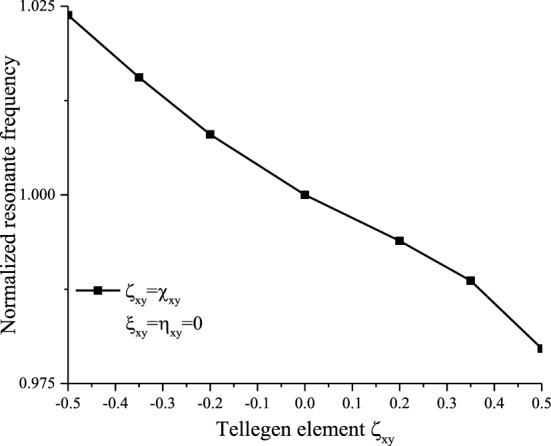
Normalized resonant frequency of a reciprocal Tellegen mediums-based dipole.

Figures 11 and 12 illustrate the effect of the bianisotropic Tellegen element on the input impedance and the resonant frequency, respectively. The values of this latter are determined from the input impedance shown in Fig. 11, from the zero crossing of the reactance curve (imaginary part)^37,38^. Figure 12 shows that the resonance frequency decreases significantly with increasing reciprocal Tellegen medium, unlike the case where the elements are purely imaginary.

### Effect of the magneto-electric parameters on the input impedance

#### Non-reciprocal achiral (${\upxi }_{{{\text{xy}}}} = - {\upeta }_{{{\text{xy}}}}$)

**Figure 13:**
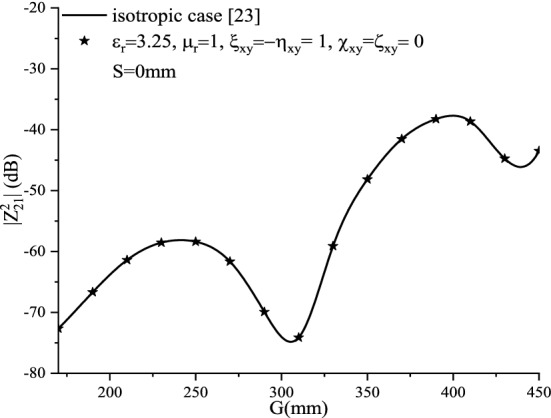
Mutual coupling of the echelon configuration of the non-reciprocal achiral elements (${\upxi }_{{{\text{xy}}}} = - {\upeta }_{{{\text{xy}}}}$).

Figure 13 shows, the mutual coupling for a non-reciprocal achiral medium case (${\upxi }_{{{\text{xy}}}} = - {\upeta }_{{{\text{xy}}}} = 1$), compared with the isotropic case. No effect is noticed on the mutual coupling.

#### Reciprocal chiral (${\upxi }_{{{\text{xy}}}} = {\upeta }_{{{\text{xy}}}}$)

**Figure 14:**
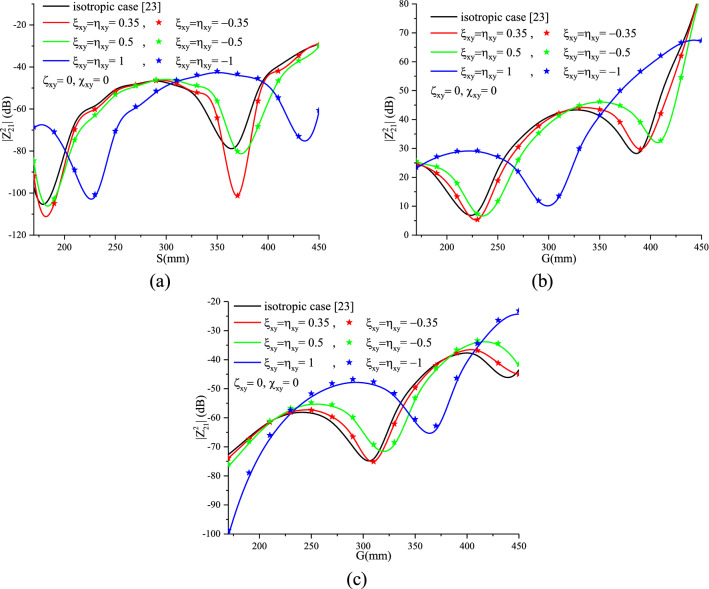
Chiral effect on the mutual coupling of an (**a**) broadside, (**b**) collinear and (**c**) echelon configuration.

Figure 14a–c show the effect of the magnetoelectric element with real, imaginary, positive and negative values on the mutual coupling of the three cases of two dipole configurations, respectively.

#### Non-reciprocal Tellegen medium ($\chi_{xy}^{{}} = - \varsigma_{xy}^{{}}$)

**Figure 15:**
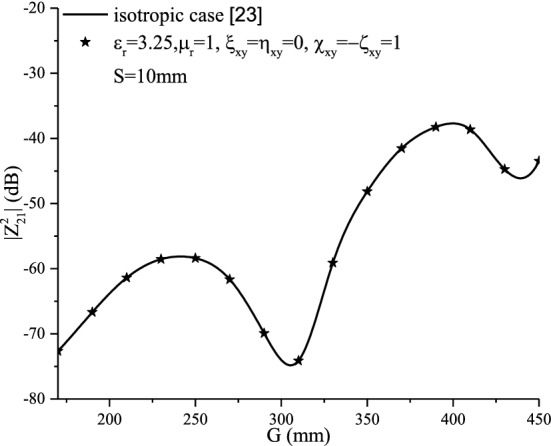
Mutual coupling of an echelon configuration with non-reciprocal ﻿bianisotropic Tellegen medium ($\chi_{xy}^{{}} = - \varsigma_{xy}^{{}}$).

In this case ($\chi_{xy}^{{}} = - \varsigma_{xy}^{{}}$), similarly to the case of non-reciprocal achiral (${\upxi }_{{{\text{xy}}}} = - {\upeta }_{{{\text{xy}}}} = 1$), it is noticed that no effect of the non-reciprocal Tellegen medium is absolutely observed on the input impedance (Fig. 10) and the mutual coupling (Fig. 15).

#### Reciprocal Tellegen medium ($\chi_{xy}^{{}}$ = $\varsigma_{xy}^{{}}$)

**Figure 16:**
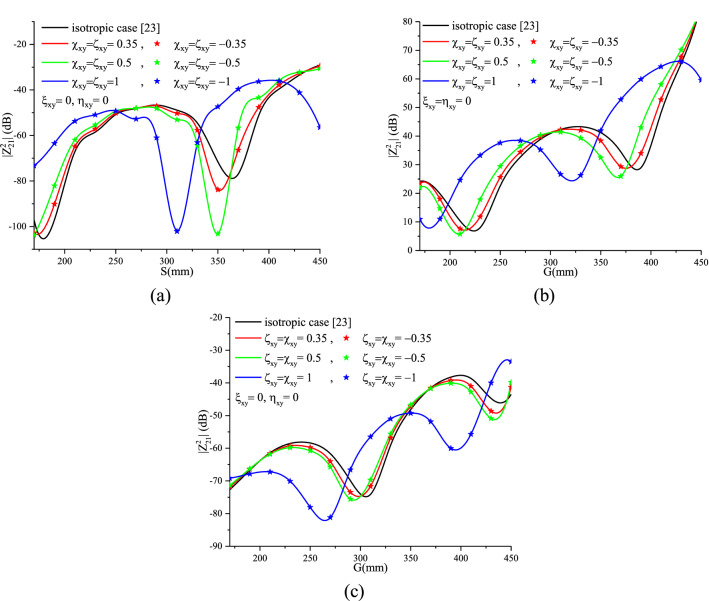
Tellegen effect on the mutual coupling of an (**a**) broadside, (**b**) collinear and (**c**) echelon configuration.

Figure 16a–c show the effect of the magnetoelectric element with real, imaginary, positive and negative values on the mutual coupling of the three cases of two dipole configurations, respectively.

From Fig. 16a–c, the Tellegen case ($\chi_{xy}^{{}}$ = $\varsigma_{xy}^{{}}$ = 0.5, $\xi_{xy}^{{}}$ = $\eta_{xy}^{{}}$ = 0) has a reciprocal behavior whereas in the case where ($\chi_{xy}^{{}}$ = $\varsigma_{xy}^{{}}$ = 0, $\xi_{xy}^{{}}$ = $\eta_{xy}^{{}}$ = 0.5) is imaginary, a slight difference between the effect of the positive and negative elements is noticed. This can be justified by exploring the equations and developed expressions elaborated by Zebiri in^8^.

A shift of the mutual coupling curves, of the three configurations, with respect to the isotropic cases, in the direction of increasing G and S is noticed for higher values of $\xi_{xy}^{{}}$ in the first studied case ($\xi_{xy}^{{}}$ is imaginary) and it is inversely in the second case ($\xi_{xy}^{{}}$ real).

The quasi-periodicity caused by surface waves increases with increasing magnetoelectric elements of the chiral medium and inversely for the Tellegen case. As an example, in the case of echelon configuration, we observe that the quasi-period equals 200 mm for the chiral element ($\xi_{xy}^{{}}$ = $\eta_{xy}^{{}}$ = 1) and 150 mm for the Tellegen element ($\chi_{xy}^{{}}$ = $\varsigma_{xy}^{{}}$).

### Effect of the substrate thickness on the mutual coupling

**Figure 17:**
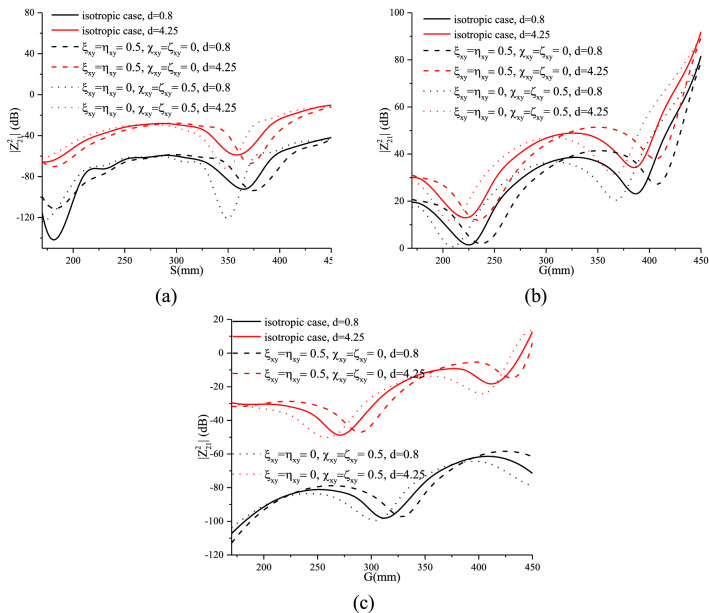
Mutual coupling for different substrate thicknesses for (**a**): broadside, (**b**): collinear and (**c**) echelon configurations.

This sub-section deals with the effect of different substrates for different values of $\chi_{xy}^{{}}$, $\varsigma_{xy}^{{}}$, $\xi_{xy}^{{}}$, $\eta_{xy}^{{}}$ and selected substrate thicknesses: 0.8 mm and 4.25 mm. Figure 17a–c illustrate the mutual coupling between the printed dipoles for these different configurations. The increase in coupling for greater substrate thicknesses is due to the increase in spatial and surface wave modes^39^. As the dipoles are spaced from the ground due to the thickness of the substrates, stronger space and surface wave modes are generated, resulting in further radiated power and more effect on parasitic elements in the vicinity of the dipoles^25^.

The beneficial choice of the medium, that must be taken into account, is that of the chiral one. As it has been shown, this latter presents a decrease in coupling of more than 30% for certain values of S or G, on the other hand, the effect of the magnetoelectric elements, whether imaginary (chiral) or real (Tellegen), keeps the same proportion with respect to the isotropic case for the two selected cases (d = 0.8 and d = 4.25).

### Effect of the magneto-electric parameters on the field’s distributions

**Figure 18:**
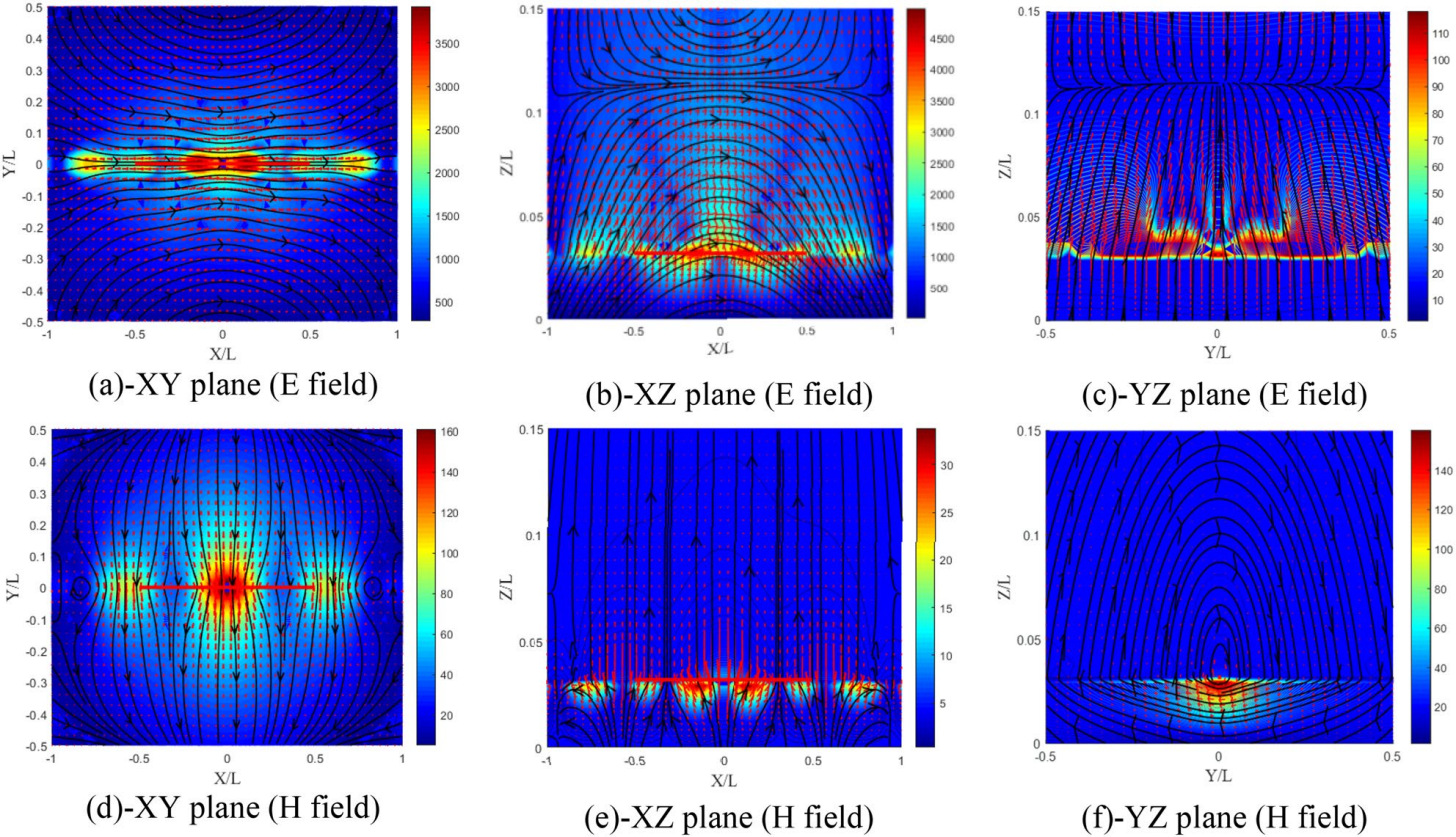
Distribution of the electromagnetic field components of the isotropic substrate (**a–c**) electric and (**d**–**f**) magnetic field components.

The electric field distributions in the XY, XZ and YZ planes are shown in Fig. 18a–c. The field distributions illustrate a conventional isotropic dipole with the XZ plane as the E plane. Similarly, the distributions of the magnetic field in the XY, XZ and YZ planes are presented in Fig. 18d,e. We observe rotating lines of the magnetic field around the dipole in the YZ plane (the H plane) according to Fig. 18e.

**Figure 19:**
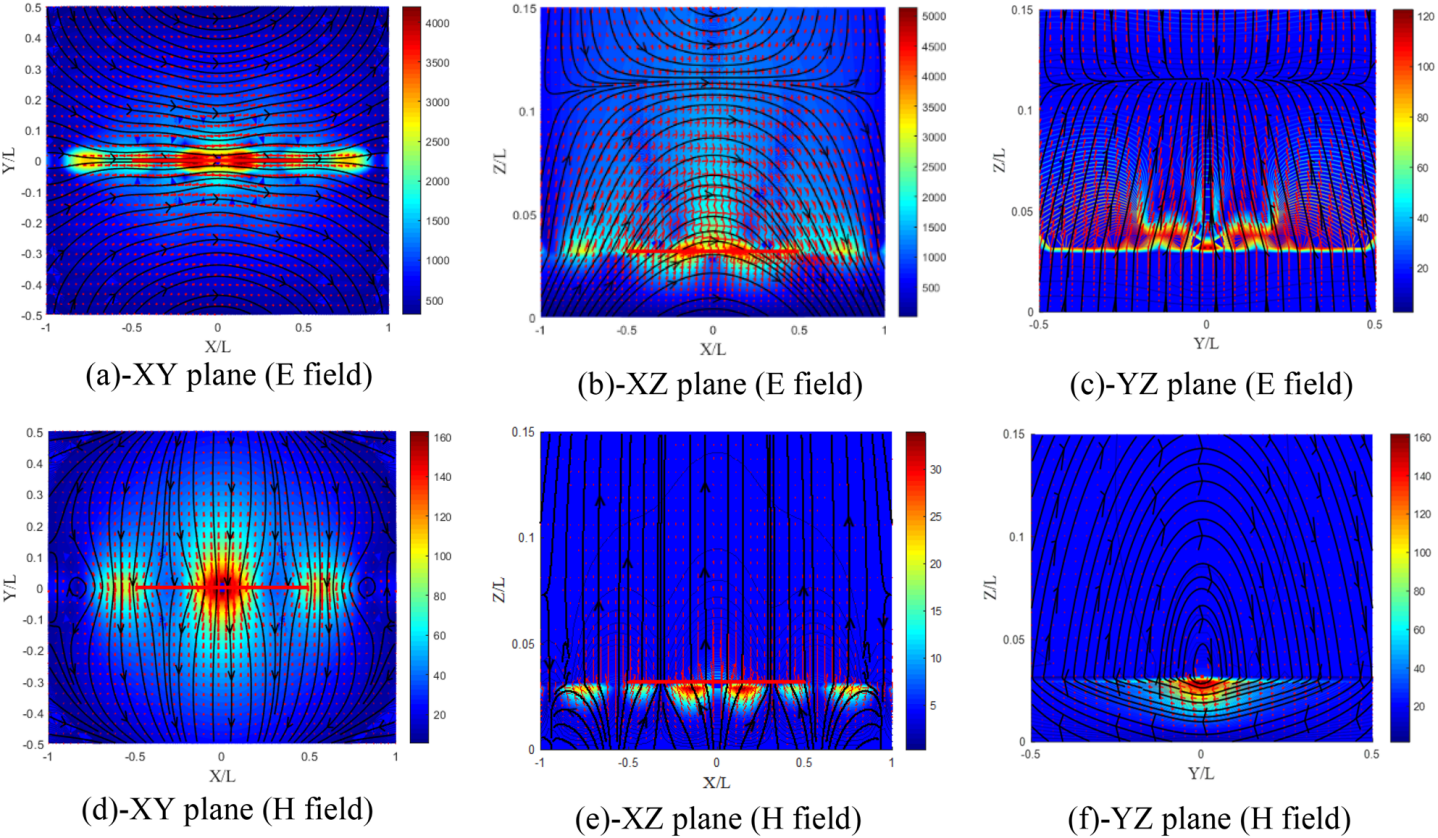
Distribution of the electromagnetic field components for (${\upxi }_{{{\text{xy}}}} = - {\upeta }_{{{\text{xy}}}}$) case, (**a**–**c**) electric and (**d**–**f**) magnetic field components.

The electric and magnetic field distributions in the XY, XZ and YZ planes are shown in Fig. 19a–f, respectively. The lines of the electromagnetic field for this case have not changed and have kept the shape of the conventional isotropic substrate dipole (Fig. 18a–f). A slight increase in the components of the electromagnetic field is noticed.

**Figure 20:**
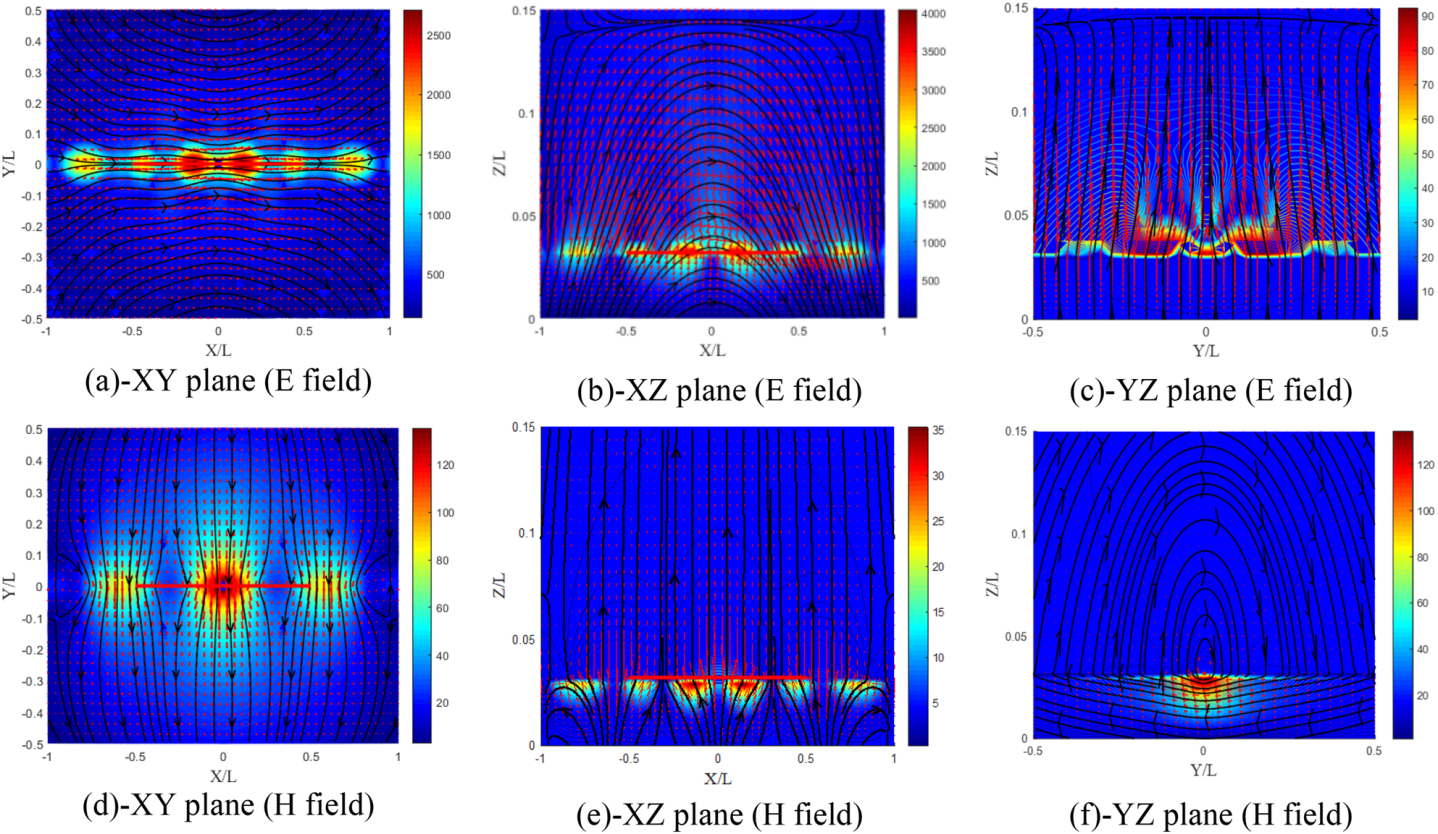
Distributions of the electromagnetic field components for (${\upxi }_{{{\text{xy}}}} = {\upeta }_{{{\text{xy}}}} = 0.5$) case, (**a**–**c**) electric and (**d**–**f**) magnetic field components.

The sign of the elements (${\upxi }_{{{\text{xy}}}} = 0.5$ and ${\upxi }_{{{\text{xy}}}} = - 0.5$) influences the distribution of the fields differently in this case. From Fig. 20a–f, for ${\upxi }_{{{\text{xy}}}} = 0.5$, we notice that the amplitude of the field components increases by 14% in the E plane with a slight decrease in the other planes. A slight decrease in the components of the magnetic field is noticed also for the three planes. The z-directed wavelength λz in this case has undergone an increase.

**Figure 21:**
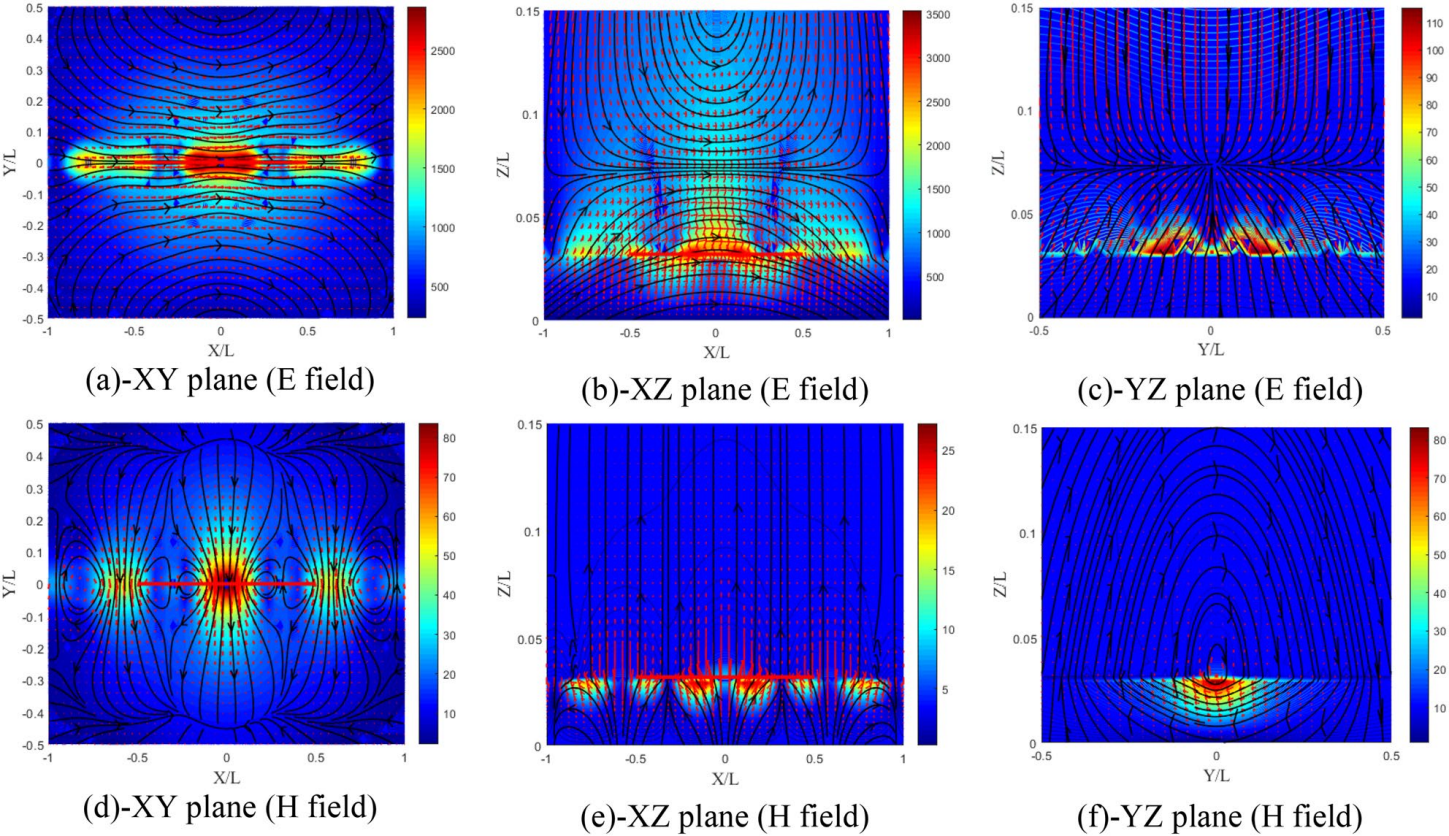
Distribution of the electromagnetic field components for (${\upxi }_{{{\text{xy}}}} = {\upeta }_{{{\text{xy}}}} = - 0.5$) case, (**a**–**c**) electric and (**d**–**f**) magnetic field components.

According to Fig. 21a–f, for ${\upxi }_{{{\text{xy}}}} = - 0.5$, we notice that the effect in this case is reversed compared to the previous case (${\upxi }_{{{\text{xy}}}} = 0.5$). The amplitude of the electric field components decreases by 24% in the E plane with a slight increase in the other planes. λz in this case experienced a decrease. However, for this case a more important decrease in the magnetic field components is noticed for the three planes.

**Figure 22:**
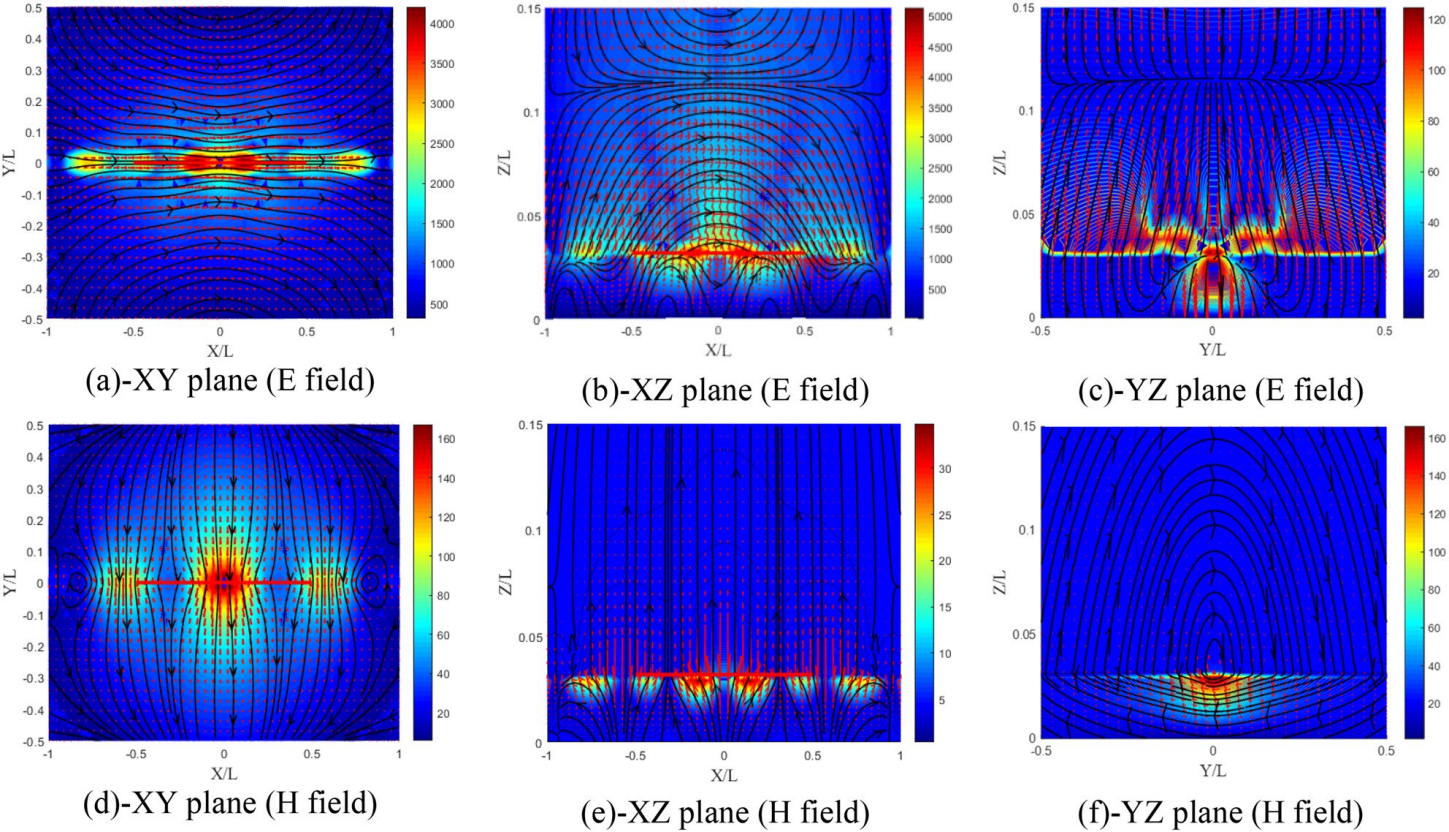
Distribution of the electromagnetic field components for (${\upxi }_{{{\text{xy}}}} = - {\upeta }_{{{\text{xy}}}}$) case, (**a**–**c**) electric and (**d**–**f**) magnetic field components.

From Fig. 22a–f, the electric field distribution in the XZ plane (Fig. 22c) is completely different from that of the isotropic case and from the previous cases, and remains unrelated because the electric field in this case is weaker compared to that in the XZ plane (E plane).

**Figure 23:**
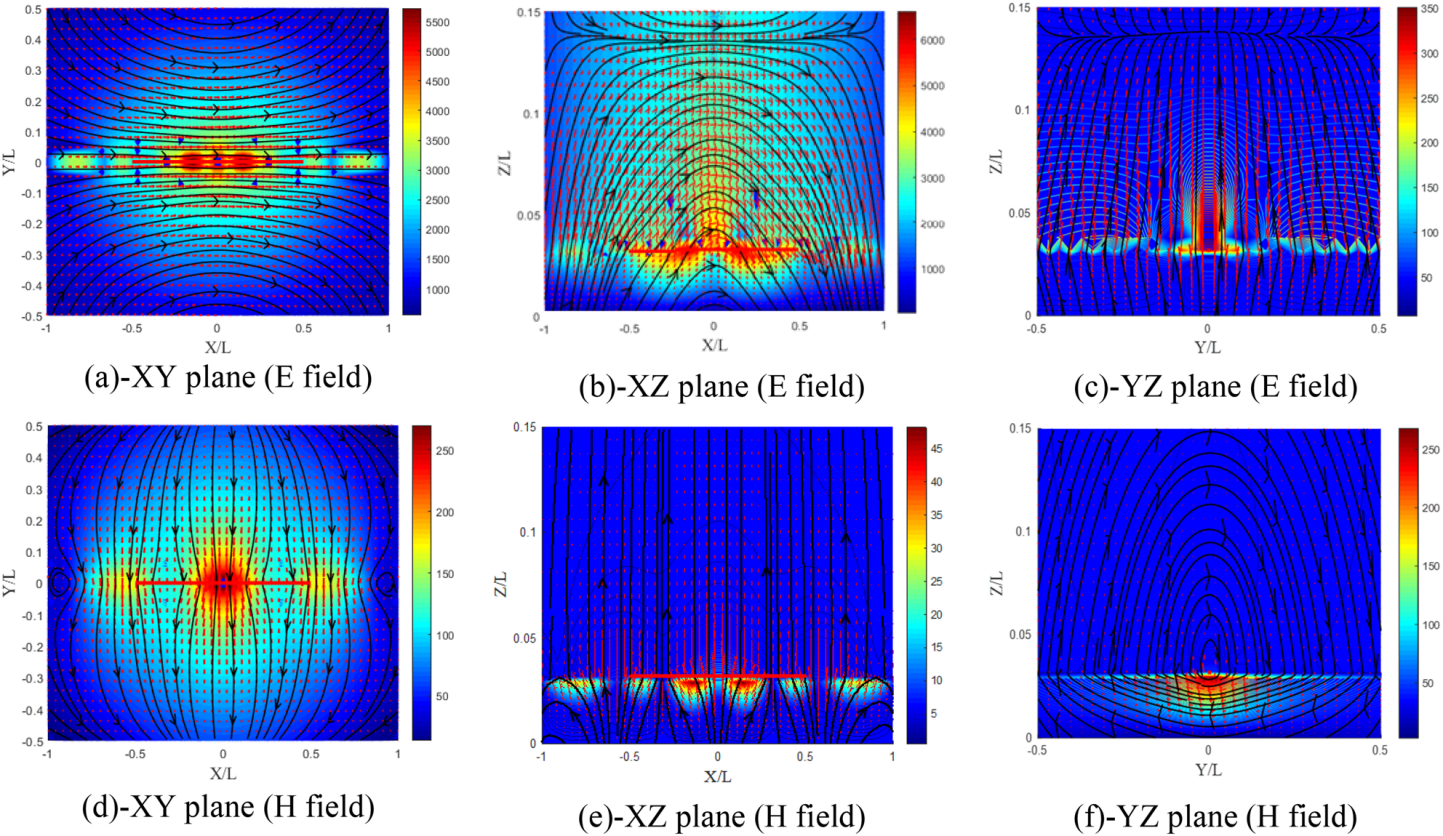
Distribution of the electromagnetic field components for ($\chi_{{{\text{xy}}}} = \varsigma_{{{\text{xy}}}} = 0.5$) case, (**a**–**c**) electric and (**d**–**f**) magnetic field components.

From Fig. 23a–f, the distribution of the electromagnetic field has kept the same shape, except that the components of the electric field have almost doubled in the E plane. Similarly, the components of the magnetic field are multiplied by about 1.7 in plane H.

**Figure 24:**
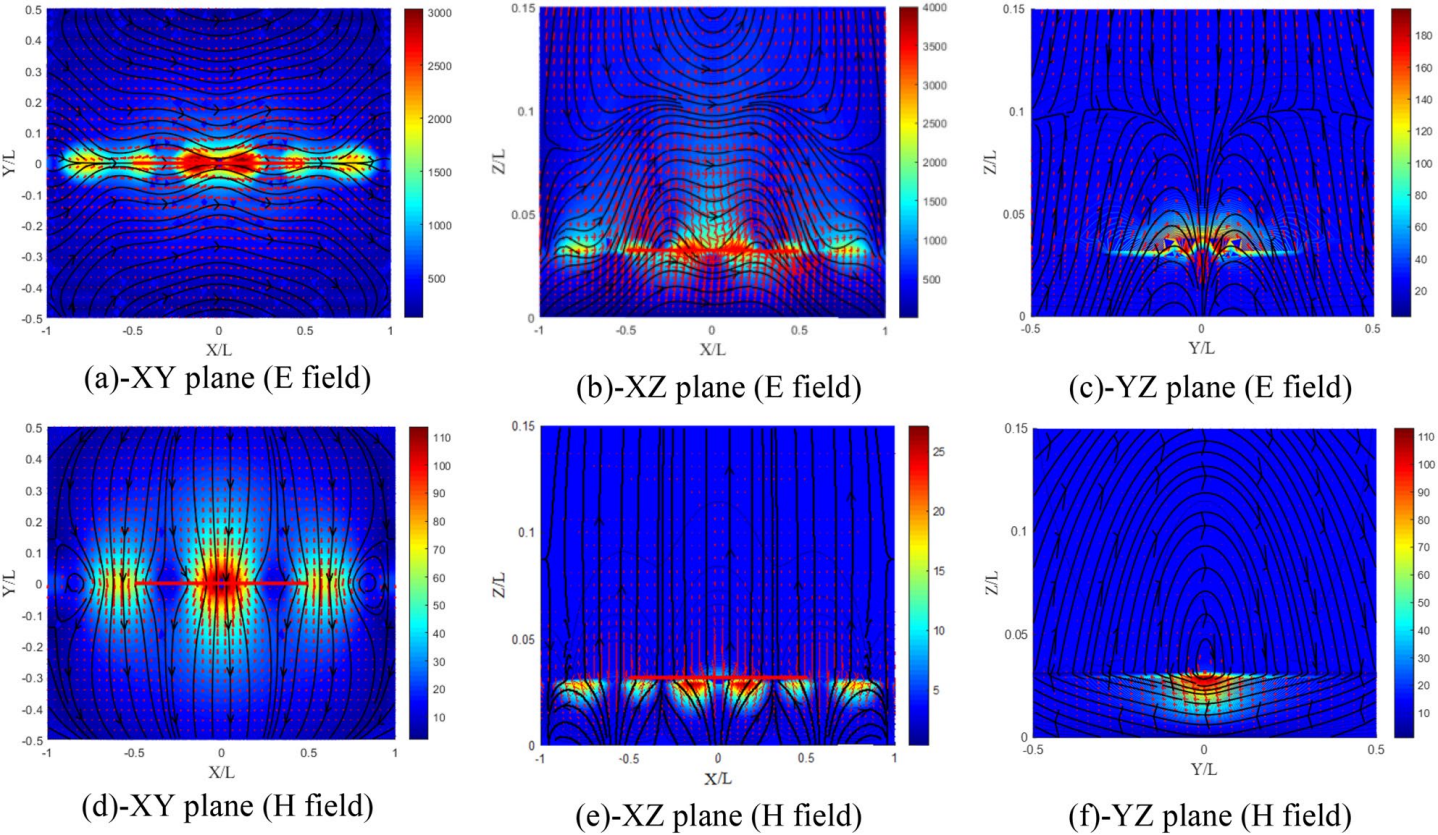
Distribution of the electromagnetic field components for ($\chi_{{{\text{xy}}}} = \varsigma_{{{\text{xy}}}} = - 0.5$) case, (**a**–**c**) electric and (**d**–**f**) magnetic field components.

For $\chi_{{{\text{xy}}}} = \varsigma_{{{\text{xy}}}} = - 0.5$ case (Fig. 24a–f), the field amplitudes are almost the same with a particular change in the distribution of the electric field in the YZ plane (Fig. 24c). The distribution has completely changed compared to the other cases and even for the isotropic case.

### Chiral and Tellegen elements advantageous combined effect on the input impedance and mutual coupling

**Figure 25:**
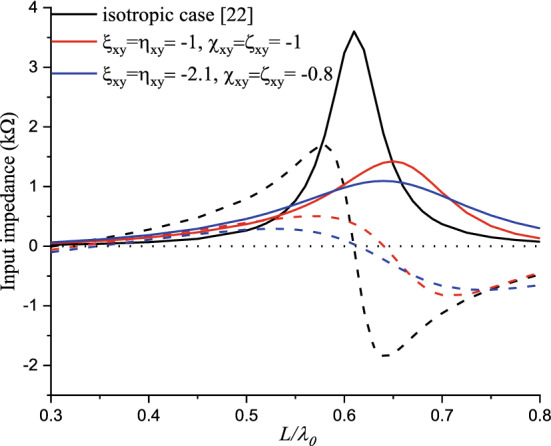
Input impedance for combined reciprocal chiral and Tellegen elements.

**Figure 26:**
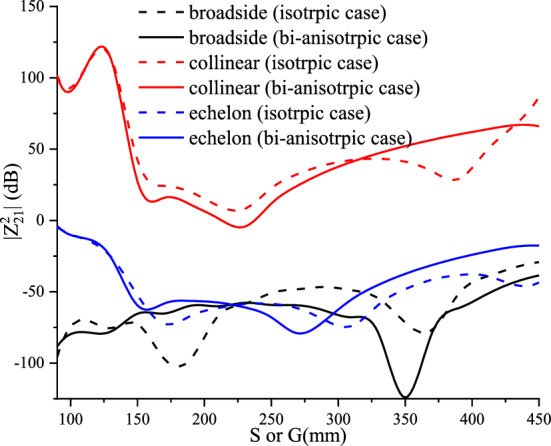
Mutual coupling for different configurations with combined chiral and Tellegen elements.

The advantageous combined effect cases of Tellegen and chiral mediums, represented by complex values of the magnetoelectric element (real and imaginary parts, respectively), on the input impedance and coupling are illustrated by Figs. 25 and 26. The selection of the elements values is based on reducing the input impedance amplitude, for matching purposes, and an improving decoupling between the antenna system elements.

The first element selection is based on the above-mentioned case ($\chi_{xy}^{{}}$ = $\varsigma_{xy}^{{}}$ = $\xi_{xy}^{{}}$ = $\eta_{xy}^{{}}$ = − 1), where a significant decrease in the peak of the input impedance accompanied with an increase in the resonant length is observed. The second selection is considered to obtain a total peak decrease of more than 75%, compared with the isotropic case, to achieve 1.02KΩ for $\chi_{xy}^{{}}$ = $\varsigma_{xy}^{{}}$ = − 0.8 and $\xi_{xy}^{{}}$ = $\eta_{xy}^{{}}$ = − 2.1, all without altering the resonant frequency value.

From Fig. 26, it is observed that the effect of the bianisotropic medium reciprocal (chiral and Tellegen) is different depending on the configuration as well as the distance between dipoles. For the collinear configuration, the chosen medium has a decoupled structure of more than 25% for the entire range between λ/4 to λ/2 (150–300 mm). For the broadside configuration, the medium shows a significant decreased mutual coupling (almost the half) for two closer dipoles (160–210 mm). For the echelon configuration, the chiral combined with the Tellegen presents an advantageous effect in the same region (160–210 mm) and above λ/2 as well. A summary of the obtained results is well described in Table 1.

**Table 1:** Summary of the obtained results.

	Values	Input impedance peak (amplitude)	Resonance frequency	Mutual coupling
$$\left[ {\upxi } \right] = - \left[ {\upeta } \right]^{T} = \left[ {\upeta } \right] = \left[ { - \begin{array}{*{20}c} 0 & {j\xi_{xy}^{{}} } & 0 \\ {j\xi_{xy}^{{}} } & 0 & 0 \\ 0 & 0 & 0 \\ \end{array} } \right]$$(Reciprocal chiral)	$\xi_{xy}^{{}} > 0$	Increase	Increase	The quasi-periodicity increases
	$\xi_{xy}^{{}} < 0$	Decrease	Decrease	The quasi-periodicity increases
$$\left[ {\upxi } \right] = \left[ {\upeta } \right]^{T} = - \left[ {\upeta } \right] = \left[ { - \begin{array}{*{20}c} 0 & {j\xi_{xy}^{{}} } & 0 \\ {j\xi_{xy}^{{}} } & 0 & 0 \\ 0 & 0 & 0 \\ \end{array} } \right]$$(Non-reciprocal achiral)	$\xi_{xy}^{{}} > 0$	No effect	No effect	No effect
	$\xi_{xy}^{{}} < 0$	No effect	No effect	No effect
$$\left[ {\upxi } \right] = - \left[ {\upeta } \right]^{T} = \left[ {\upeta } \right] = \left[ { - \begin{array}{*{20}c} 0 & {\chi_{xy}^{{}} } & 0 \\ {\chi_{xy}^{{}} } & 0 & 0 \\ 0 & 0 & 0 \\ \end{array} } \right]$$(Reciprocal Tellegen 1^st^ case)	$\chi_{xy}^{{}} > 0$	Increase	Decrease	The quasi-periodicity decreases
	$\chi_{xy}^{{}} < 0$	Decrease	Increase	The quasi-periodicity decreases
$$\left[ {\upxi } \right] = \left[ {\upeta } \right]^{T} = - \left[ {\upeta } \right] = \left[ { - \begin{array}{*{20}c} 0 & {\chi_{xy}^{{}} } & 0 \\ {\chi_{xy}^{{}} } & 0 & 0 \\ 0 & 0 & 0 \\ \end{array} } \right]$$(Non-reciprocal Tellegen 2^nd^ case)	$\chi_{xy}^{{}} > 0$	No effect	No effect	No effect
	$\chi_{xy}^{{}} < 0$	No effect	No effect	No effect
$$\left[ {\upxi } \right] = - \left[ {\upeta } \right]^{T} = \left[ {\upeta } \right] = \left[ { - \begin{array}{*{20}c} 0 & {\left( {\chi_{xy}^{{}} + j\xi_{xy}^{{}} } \right)} & 0 \\ {\left( {\chi_{xy}^{{}} + j\xi_{xy}^{{}} } \right)} & 0 & 0 \\ 0 & 0 & 0 \\ \end{array} } \right]$$(Reciprocal complex bianisotropic)	$\xi_{xy}^{{}} = - 1$ and $\chi_{xy}^{{}} = - 1$	Decrease < 60%	Increase > 10%	–
	$\xi_{xy}^{{}} = - 2.1$ and $\chi_{xy}^{{}} = - 0.8$	Decrease < 75%	Increase > 2%	The quasi-periodicity decreases by more than 25%

## Conclusions

In this paper, a rigorous mathematical formulation describing the bianisotropic medium electromagnetic behavior is presented and the input impedance, the electromagnetic field distributions and the mutual coupling in two-element printed dipole array are investigated. The field distributions in such a medium will be the start of an in-depth study of the bianisotropic medium behavior to better understand the effects of this medium on different parameters of the dipole antenna, for which some have been shown here. The medium bianisotropy effect on the input impedance of a single dipole configuration is evaluated. It is shown that, with increasing magnetoelectric elements of the chiral medium, the quasi-periodicity caused by surface waves increases and inversely for the Tellegen medium. An appropriate selection of the magnetoelectric elements, combined effect of chiral and Tellegen mediums, leads to a significant decrease in the input impedance. A peak decrease of more than 75% is achieved without altering the resonance frequency, which is advantageous for matching possibilities. It is also concluded that this choice leads to a better decoupling compared with the isotropic case. An improvement of 25% up to 200% in the case of the broadside configuration for small distances between dipoles (between λ/4 and λ/3) is obtained.
